# Emerging neurotechnological approaches to management of sleep disturbances in Parkinson’s disease

**DOI:** 10.1016/j.prdoa.2025.100413

**Published:** 2025-12-13

**Authors:** Mason Dallegge, Sanjana Murthy, Don M. Tucker, Emmanuel During, Shannon O’Neill, Rachel Fremont, Joohi Jimenez-Shahed, Allison C. Waters

**Affiliations:** a Icahn School of Medicine at Mount Sinai, Department of Neuroscience, USA; b Icahn School of Medicine at Mount Sinai, Department of Psychiatry, USA; c Brain Electrophysiology Laboratory Company, University of Oregon, USA; d Icahn School of Medicine at Mount Sinai, Department of Neurology, USA; e Icahn School of Medicine at Mount Sinai, Department of Neurosurgery, USA

## Abstract

Among non-motor symptoms of Parkinson’s disease (PD), sleep disruption is highly prevalent and may be a target for emerging neurotechnological approaches to treatment. Neuroanatomical and chemical changes in PD significantly impact sleep regulation, affecting both REM and non-REM sleep stages. These disturbances contribute to cognitive decline and disease progression, underscoring the critical need for effective management strategies. Sleep-focused neurotechnological advancements offer promising avenues for enhancing treatment of PD. Cognitive Behavioral Therapy for Insomnia (CBT-I) successfully addresses maladaptive sleep behaviors exacerbated by PD and may be further enhanced with the use of sleep tracking technologies. At-home EEG-based monitoring complements CBT-I by facilitating real-time adjustments and optimizations and provides insights into personalized sleep management strategies. Additionally, neuromodulation techniques using direct stimulation aim to restore deep sleep (N3) by targeting specific brain regions affected by PD-related neurodegeneration. This article reviews the presentation of sleep disturbances in PD, explores the potential role for physiological tracking and neuromodulation in treatment of PD, and explores the use of consumer technologies to support personalized sleep management for patients and clinicians.

## Highlights

• Disrupted sleep architecture is a contributing factor to disease progression in Parkinson’s Disease (PD)
• An unmet treatment need exists in the current clinical approach to the treatment of sleep dysfunction in PD.
• Neurotechnological approaches offer novel therapeutic avenues for treatment of sleep disorders.
• Targeted enhancement of deep sleep in PD may slow or ameliorate disease progression.

## 1 Manifestations of disordered sleep in Parkinson’s disease

Parkinson’s disease (PD) is a neurodegenerative disease characterized by the loss of midbrain dopaminergic neurons, particularly within the substantia nigra. The presentation of PD is varied, but most commonly manifests as a combination of both motor and non-motor symptoms. Disordered sleep is one of the most prevalent non-motor symptoms of PD, with as high as 90 % of patients reporting nocturnal sleep disruptions [1]. Those suffering commonly complain of difficulty falling asleep and nighttime awakenings, contributing to fragmented and inefficient sleep [2]. The underlying cause of these sleep disruptions is multifactorial in nature and contributes to significant changes of sleep architecture, the cyclical neurophysiological activity that composes nocturnal sleep stages. Slow wave sleep (SWS) and Rapid Eye Movement (REM) sleep are often degraded in PD yet are essential for memory consolidation, cognitive function, and the clearance of metabolic debris. REM sleep behavior disorder (RBD) is also commonly observed within PD, often further disrupting normal sleep patterns [3]. Degraded sleep is known to be associated with worse outcomes in PD and may also increase the rate of disease progression [4]. Disrupted sleep architecture is also associated with excessive daytime sleepiness in PD, leading to increased napping or dozing during the day. This impacts quality of life and further exacerbates problems with nocturnal sleep regulation. Interrupting this cycle between disrupted sleep and disease severity is a critical target to improve daily functioning and slow the trajectory of PD.

### 1.1 Bidirectional impact of sleep disturbances and PD

Sleep health and the progression of PD are deeply intertwined, where continued neurodegenerative change is reflected in both motor symptomatology and sleep physiology. A positive feedback loop exists for many patients with PD suffering from sleep disorders, where loss of sleep homeostasis both originates from and further contributes to PD progression [5]. Crucial physiological processes, such as glymphatic debris clearance and memory consolidation are implicated in the degradation of sleep health, precipitating further disease progression [6]. Unsurprisingly, health outcomes are profoundly impacted by the presence of sleep dysfunction in PD. Impaired sleep quality in PD has been linked to worse quality of life, worse cognition, and poorer motor function [7], [8]. Furthermore, poor sleep quality predicts PD development and progression [1], [9]. To understand how these reciprocal effects arise, it is essential to examine the underlying drivers of both sleep dysregulation and neurodegenerative processes.

Sleep disruption in PD is associated with numerous causal factors. Pharmaceutical management is of key importance in this regard, as dopaminergic interventions have significant influence on the maintenance of wakefulness and regular circadian rhythms [10], [11]. Administration of dopamine therapeutics can heavily impact sleep regulation, where inadequate nocturnal coverage of drugs such as levodopa can contribute to akinesia [10]. Simultaneously, over-administration of dopamine agonists or precursors may promote somnolence or cause sleep attacks, further contributing to sleep deregulation [10]. The management of these therapeutics is particularly important, as it also addresses another concern for sleep disruption, nocturnal motor symptoms. In clinical populations, roughly 60 % of patients experience motor symptoms leading to disrupted sleep [12]. These symptoms may include rigidity, tremor, restless leg syndrome, and others.

Patient age is also a key factor in PD sleep disruptions, as advanced age is an established risk factor for both PD and insomnia [13], [14]. Aging is associated with natural changes to sleep architecture, which may include circadian disruption, more frequent nocturnal awakenings, and earlier awakenings [14]. Another important consideration in PD pertains to sleep disordered breathing, including conditions such as obstructive sleep apnea. While results are divided on whether obstructive sleep apnea appears in PD at a higher rate than the general population, recent findings suggest that if left untreated, it may actually increase the risk of developing PD several fold [15], [16]. Moreover, sleep disordered breathing can significantly impair sleep health in patients with PD. Deoxygenation events overnight can further contribute to overnight awakenings and disruptions in sleep architecture, leading to reductions in sleep quality [17].

Often, sleep degradation mirrors progression of other symptoms of PD, including motor and executive function [4]. This may suggest an interrelated neurodegenerative pathway between early synucleinopathy and the incidence of disordered sleep. Prodromal sleep-related symptoms of PD, which often occur years prior to the development of motor symptoms, could reflect neurodegenerative change preceding the degeneration of nigrostriatal pathways. As patients progress to later stages of disease, they often display severe degradation of sleep, where destructuring of sleep architecture is the most pronounced and most debilitating [18].

### 1.2 Altered circadian rhythm

**Fig. 1:**
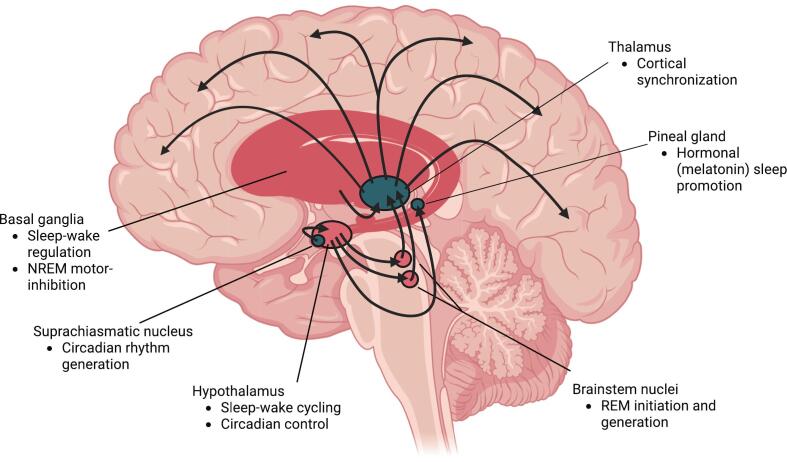
**Disruption of sleep-regulating neuroanatomy in Parkinson’s disease.** Brain structures associated with Parkinson’s disease (pink) reflect progressive neurodegeneration in function critical for regulating sleep. Early degeneration in brainstem nuclei contributes to REM sleep disruption, while damage to basal ganglia and thalamocortical loops impairs slow-wave sleep. Circadian signaling is further disturbed through degradation of the suprachiasmatic nucleus (SCN) and impaired melatonin release from the pineal gland. These changes result in fragmented and poorly structured sleep cycles, contributing to daytime dysfunction and potential acceleration of disease progression. Created in BioRender.com. (For interpretation of the references to colour in this figure legend, the reader is referred to the web version of this article.)

Sleep-wake cycles can be severely altered in PD, contributing to both waking and nocturnal disruptions. Insomnia is the most common sleep disorder in PD, affecting up to 80 % of all patients with PD [19]. Insomnia is characterized by difficulty falling asleep, difficulty maintaining sleep, or early-morning wakefulness with an inability to return to sleep and is also associated with daytime impairment[20]. Excessive daytime sleepiness describes pronounced drowsiness and inability to maintain arousal during waking hours [21]. In many patients, this leads to excessive dozing or napping during the day, and may contribute to “sleep attacks”, or a sudden onset of sleep during wake [21]. Neurodegeneration to sleep-wake centers of the brain (see Fig. 1), the effect of medications, and sociobehavioral aspects may all play a role in these symptoms. Degeneration of the suprachiasmatic nucleus appears to disrupt downstream signaling to the pineal gland, the primary site for melatonin generation [22]. This disruption likely contributes to the blunted melatonin rhythm observed in PD [23], further altering normal circadian rhythm.

### 1.3 REM sleep behavior disorder

Perhaps the most striking sleep disturbance in Parkinson’s Disease and other synucleinopathies is RBD, in which the motor inhibition during REM sleep is disrupted, allowing disturbing and potentially dangerous motor behaviors to be carried out during the dream state [3], [24]. Evidence suggests that RBD is due to neural degeneration in the brainstem circuits that normally inhibit behavioral engagement associated with the internal activity of the REM sleep process [24]. RBD is highly prevalent within the PD population [3], experienced by 15–47 percent of patients, and contributes to nocturnal awakenings and disruption of the sleep cycle. REM sleep is implicated in the consolidation of procedural and emotional memory, and its dysfunction in PD may further impair cognitive function [25].

REM sleep is largely governed by brainstem structures, including the pedunculopontine tegmental nucleus and the laterodorsal tegmentum [26]. The ascending theory of PD, described by Braak in 2003, suggests that early neurodegeneration involves the brainstem nuclei, before it spreads to the substantial nigra, limbic system and higher cortical areas [27]. This is further substantiated by the incidence of RBD symptoms in many cases more than one decade before the onset of motor symptoms in PD [28], [29].

Disruptions of REM sleep [30], including RBD, also impact the glymphatic system, a waste clearance pathway that uses cerebrospinal fluid to flush out metabolic debris during deep sleep. Disruptions to sleep homeostasis impair glymphatic function, and thereby the clearance of toxic metabolic byproducts [31]. The primary marker of neurodegeneration in PD is the presence of Lewy bodies, the build-up of misfolded alpha-synuclein, a neuronal protein involved in synaptic function. The accumulation of misfolded proteins in these toxic clumps has be attributed in part to impaired performance of the glymphatic system [32], [33]. Clarifying the link between Lewy body pathology, the presence of RBD and glymphatic dysfunction is an active area of research. One emerging hypothesis is that targeted restoration of glymphatic function could support the clearance of pathological debris, potentially reducing the severity of PD.

### 1.4 Changes in sleep architecture

In healthy sleep, the brain cycles between Non-Rapid Eye Movement (NREM) and Rapid Eye Movement (REM) sleep. NREM sleep predominates in earlier hours of sleep, with REM proportionally increasing as the night continues. In PD, this rhythm is disrupted, as motor symptoms and pathological neural activity contribute to intermittent nocturnal awakenings [34]. This leads to a fragmented presentation of sleep architecture as the number of arousals and sustained wake periods increases, where the deepest stage of NREM sleep (SWS or N3) and REM sleep periods may only be sustained for minutes at a time [35]. Of note, sleep continuity and cycling through sleep stages is also affected by typical aging, in which sleep time shifts towards the lighter non-REM sleep stages, N1 and N2 [13]. These changes, in combination with PD-related degradation of sleep stages, leaves older PD patients at particular risk for a relative deficit in deep, restorative sleep.

Although REM sleep disturbances such as RBD are central to understanding sleep neuropathology in PD, reductions in NREM sleep are also concerning [10], [36]. NREM sleep is host to important neurophysiological features such as sleep spindles, which have been linked to declarative memory consolidation and synaptic regulation [37], [38]. NREM sleep is primarily regulated by structures of the basal ganglia and brainstem [39]. In PD, degeneration of dopaminergic neurons within the basal ganglia, specifically the substantia nigra pars compacta, precipitate the distinctive motor symptoms of the disease [40]. Downstream structures from these neurons are understood to promote cortical synchronization during sleep, which may contribute to the nocturnal disruptions observed in PD [39]. Other structures involved in the regulation of healthy sleep, such as the thalamus and hypothalamus, also undergo neurodegenerative changes [40]. The degradation of these structures likely contributes to circadian rhythm disorder and impaired regulation of wakefulness and sleep [41].

The progression of NREM and REM stages in each sleep cycle implies a causal relation between these stages; NREM intervals are extended in the early cycles of the night, with only brief REM intervals, while later cycles show decreasing NREM and increasing REM sleep duration. This suggests that the neurophysiological mechanisms of NREM sleep may be necessary before REM sleep is permitted to become the dominant sleep stage [42]. It follows that the most prominent manifestations of sleep dysfunction in PD, such as RBD, may be closely linked to NREM degeneration. Through effective restoration of deep, NREM sleep, it may be possible to achieve therapeutic benefit on a wider scope of sleep disturbances in PD.

## 2 Emerging neurotechnological treatment for Parkinson’s disease

Current management of disordered sleep in PD hinges largely upon pharmacological and behavioral approaches. The frontline non-pharmacological treatment for insomnia is cognitive behavioral therapy for insomnia (CBT-I), which aims to address the disrupted processes involved in maintaining insomnia, such as dysregulation of sleep drive, sleep-related anxiety, and sleep-interfering behaviors [43]. However, access to CBT-I specialists with knowledge of PD is limited, and not all patients with PD respond to CBT-I at the same rate [44], necessitating new approaches to enhance access and outcomes of behavioral treatment.

Neurotechnology, including sleep monitoring devices and neuromodulation approaches, may offer a unique advantage in the treatment of sleep dysfunction in PD. The consumer space surrounding sleep health is rapidly expanding, with numerous commercially available products existing. Health trackers (Table 1), such as Fitbit and Oura Ring, offer sleep staging information while continuous advancement is being made in both invasive and non-invasive sleep neuromodulation (Table 2). The data and implications to patient care offered by both types of technology are intriguing and may offer unique avenues to the enhancement of treatments like CBT-I.

**Table 1:** Commercial sleep tracking.

**Device Type**	**Examples**	**Sleep Staging Metrics**	**Pros**	**Cons**
Wrist Wearables	FitBit, Garmin, Apple Watch, Whoop	Movement and heart rate	Ease of use, integration with other devices	Sleep versus wake validity
Rings	Oura Ring, Galaxy Ring	Movement and heart rate	Ease of use, comfort	Sleep versus wake validity
Mattress Covers	Eight Sleep Pod, Nitetronic G1, Sleepme Sleep Tracker	Movement, respiration, and heart rate	Low profile, ease of use	Price, accuracy
EEG Wearables	Muse Headband, Dreem Headband	EEG, heartrate, and movement	Accuracy, neural insights	Availability

**Table 2:** Sleep neuromodulation landscape.

**Device**	**Manufacturer**	**Sleep Staging Method**	**Stimulation Method**	**Indication**	**Commercial Availability**
Sleep WISP	Brain Electrophysiology Lab (BEL)	EEG	Transcranial alternating current	Insomnia	RCT ongoing
Elemind Headband	Elemind	EEG	Acoustic	Insomnia	Available
Inspire Implant	Inspire	N/A	Hypoglossal Nerve	Obstructive sleep apnea	Available
Modius Sleep	Neurovalens	N/A	Vestibular Nerve	Insomnia	RCT Ongoing

### 2.1 Technology enhanced cognitive behavioral therapy for insomnia

CBT-I is comprised of two main behavioral interventions, stimulus control and sleep restriction. Stimulus control strategically targets the learned association between the bed and sleep by encouraging usage of the bed only for sleep and intimacy. Sleep restriction increases sleep drive by limiting the amount of time spent in bed not sleeping to match actual sleep duration, thereby increasing sleep efficiency and reducing anxiety over insomnia. Cognitive restructuring is also central to CBT-I delivery, targeted at changing maladaptive perceptions or beliefs about sleep. Reinforcement is often provided for good sleep hygiene and engagement in relaxing behaviors, including meditation and mindfulness, that may help limit cognitive arousal and promote sleep [45]. Many of these components of CBT-I mirror those used in alternative or traditional medicine practices, which have also shown promise in the treatment of non-motor PD symptoms [46]. Most CBT-I activities rely on the expertise of the facilitator and the accuracy of the sleep diary, a patient-completed measure used to monitor sleep characteristics such as bedtime, waketime, overnight awakenings, and more. Both represent opportunities for augmentation with technology.

Studies of CBT-I in PD have shown it to be successful in reducing nocturnal total wake time, increasing sleep efficiency, and improving subjective sleep quality [44], [47]. However, limited access to providers of CBT-I creates an obstacle for those seeking this manner of care. Mobile applications, such as “CBT-i Coach” [48], seek to remedy this by providing an accessible platform for self-guided CBT-I. Previous studies suggest that these applications may be as effective as traditional CBT-I [49], though individuals that are unfamiliar or uncomfortable using technology may find them challenging to use. Furthermore, advancements in artificial intelligence (AI) based CBT-I instruction are increasing the availability amongst the general population without sacrificing protocol individualization provided by expert facilitators [50]. When combined, biometric sleep tracking devices and AI CBT-I instruction may be a promising approach to lower the barrier of entry for personalized sleep care in PD.

### 2.2 Self-monitoring of sleep physiology

Given the progressive nature of sleep disruption in PD, accurate and efficient monitoring of sleep symptomatology is critical to inform treatment. The gold-standard method for sleep monitoring and assessment is video-polysomnography (PSG) [51], which generally involves a single-night recording of neural and physiological activity in a clinical sleep laboratory. However, its limited practicality highlights the need for convenient, affordable, home-based alternatives that can support large-scale use and enable long-term sleep monitoring. At-home polysomnography can be achieved using portable polysomnography devices, however the accuracy of these devices falls short of studies conducted in a sleep laboratory [52]. Behavioral interventions, such as CBT-I, rely upon patient report or manual entry into a sleep diary. However, this method can be prone to bias due to inaccuracies or subjectivity in patient reporting. Moreover, the range of sleep pathologies that can be diagnosed in this manner is limited in the absence of objective information. To support effective, longitudinal diagnosis and treatment of disordered sleep in PD, sleep monitoring approaches must balance accuracy with feasibility of long-term patient use.

Wrist-worn actigraphy, or similar activity-based monitoring devices, have recently gained considerable attention in sleep research and clinical practice [53]. Data collected by such devices provide an accurate approach to sleep tracking, while being simultaneously low-burden and well suited for long-term use. Among the general population, commercial sleep tracker use is significant, with roughly one third of individuals reporting use of a wearable device or mobile application [54]. Wearable devices, mattress covers, and sleep pads utilize combinations of movement, heart rate, body temperature, and respiration to estimate sleep stages. Sleep tracking apps are also numerous, incorporating user input or sound-based data to provide insights into sleep health. It is important to acknowledge that the accuracy of this data, particularly that of sleep staging, falls short of that provided by polysomnography, especially in non-healthy populations [55], [56], [57].

EEG-guided sleep staging offers distinct advantages over many common commercially available sleep trackers by providing real-time, objective, neural measures. EEG can identify CBT-I-related changes in sleep patterns, such as improvements in slow-wave activity, sleep latency, and wake after sleep onset [58]. While EEG capable sleep staging devices are less commonly available to consumers, they can serve as an objective measure of treatment response by providing immediate insight into changes in sleep architecture and sleep stage distribution. Data collected in this manner is capable of supplementing subjective reports of sleep quality, thereby helping to validate the efficacy of CBT-I across different patient populations [44], [59], [60]. At-home EEG sleep monitoring faces challenges, such as reductions in signal quality from disrupted skin electrode contact or complications in data processing. However, AI-driven sleep staging algorithms may mitigate these issues.

An important consideration when discussing at-home sleep tracking is the prevalence of orthosomnia, or the preoccupation with improving or perfecting sleep quality [61]. This phenomenon is most commonly observed in sleep medicine clinics, where patients exhibit obsessive behaviors in the pursuit of better sleep, resulting in increased anxiety and even worsening of insomnia symptoms. As such, it is important that realistic expectations are set when it comes to the incorporation of sleep tracking devices and software when treating disordered sleep. Furthermore, it is crucial that further research is conducted to assess the impact and extent of these metrics on patient care.

### 2.3 Sleep neuromodulation

Sleep neuromodulation is an intriguing and emerging prospect for the treatment of sleep disorder in PD. Ongoing clinical trials seek to understand the role of various approaches to neuromodulation as potential therapeutic options for disordered sleep. As the understanding of both sleep physiology and the potential role of neuromodulation in this space progress, it is crucial that future studies fully explore how these approaches may therapeutically suit sleep dysfunction in PD. Approaches to sleep neuromodulation are varied but primarily consist of non-invasive stimuli applied directly prior to or during sleep. PD, as well as other neurodegenerative diseases, stand to benefit greatly from interventions that are capable of addressing the underlying causes of sleep dysfunction.

**Fig. 2:**
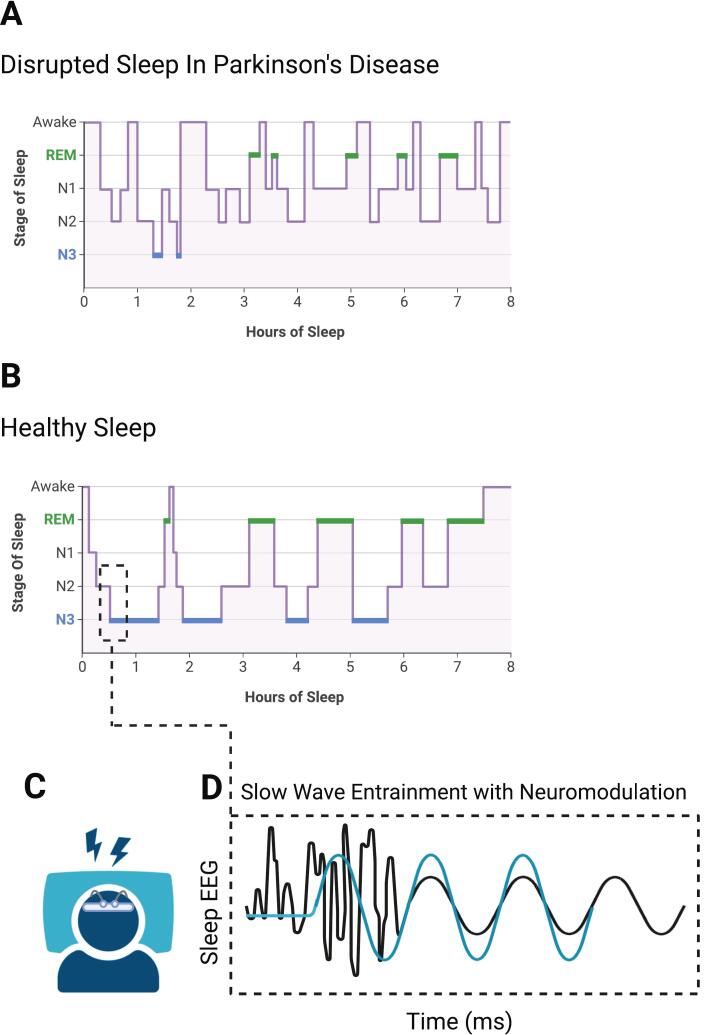
**Proposed mechanism of slow wave sleep enhancement with sleep neuromodulation in PD.** Panel A depicts a sleep hypnogram that may be observed in Parkinson’s disease (PD). Sleep stages are fragmented and sleep continuity is disrupted, interfering with normal restorative processes. This is in contrast with the typical progression of sleep cycles, depicted in panel B. Non-invasive neuromodulation techniques such as transcranial electrical stimulation (C) and phase-locked auditory stimulation aim to entrain endogenous slow oscillations (D). Stabilizing SWS through entrainment may reinforce REM stage stability, enhance glymphatic clearance, support memory consolidation, and improve sleep-dependent neurophysiology in PD. Created in BioRender.com.

The therapeutic effect of neuromodulation hinges directly on the selection of stimulation parameters. Key considerations include stimulation frequency, amplitude, and phase of stimulation. For example, low-frequency, phase-locked stimulation during NREM sleep is suggested to synchronize cortical oscillations (see Fig. 2), while down-phase stimulation can disrupt neural activity and contribute to sleep fragmentation [62]. Target selection further dictates physiological effect, as stimulation of different cortical areas can influence sleep architecture in unique ways. Recent efforts to optimize target identification for neuromodulation have shed light on the cortical sources of key sleep features and represent a push for optimized stimulation parameters in sleep neuromodulation [63].

### 2.4 Transcranial electrical stimulation

Transcranial electrical stimulation (TES) involves the application of current, typically 0.5–2 mA, which is thought to modulate cortical electrophysiology during or directly prior to sleep [64]. Sleep TES is primarily administered via one of two modalities: transcranial direct current stimulation (t-DCS) or transcranial alternating current stimulation (t-ACS). Studies of t-DCS in non-PD populations have suggested that the application of direct current can directly modulate cortical excitability [65], which may be capable of lowering the threshold required for activation of key sleep promoting centers. The use of excitatory t-DCS has been shown to enhance intrinsic sleep characteristics such as total sleep time and memory consolidation [66], [67], which may be a viable approach in PD. Previous studies have shown observable effects after just 1–5 nights of stimulation. Existing t-DCS systems include the Neuroelectrics Starstim (Neuroelectrics, Barcelona, Spain) and Soterix tDCS (Soterix Medical, Woodbridge, NJ), though both are currently intended solely for research or clinical applications and are not commercially available to the general public. Products such as the Flow Neuroscience Flow Headset (Flow Neuroscience, Malmö, Sweden) are commercially available but have not yet been examined in the context of sleep.

T-ACS has also been examined for its potential to modulate sleep, though research is not as comprehensive as with t-DCS. T-ACS involves the application of a sinusoidal electrical stimulus targeting endogenous neural oscillations [68]. This is understood to entrain neural activity [69], [70], which may act as a stabilizing mechanism for slow-wave activity during sleep [71]. These characteristics may offer a unique benefit to sleep in PD by synchronizing and supporting the often fragmented presentation of slow oscillations in SWS. Current t-ACS capable products for sleep neuromodulation include the BEL Sleep WISP (Brain Electrophysiology Lab, Eugene, OR) and Somnee Smart Sleep Headband (Somnee, Berkeley, CA), of which the latter is currently commercially available. Previous studies report that singular, brief stimulation sessions administered early in the night are capable of increasing slow-wave sleep duration by roughly 7 % [63].

Both t-DCS and t-ACS as neuromodulative approaches for the improvement of sleep necessitate further research, however, as there have been conflicting results on their efficacy [72], [73]. For example, previous studies have suggested that low-frequency stimulation does not entrain slow oscillations during sleep [72], raising questions about the mechanism of action, and that overnight t-DCS may be inconsistent in improving memory consolidation [73]. The feasibility and efficacy of these modalities must be established in both healthy individuals and populations with neurodegenerative disease, where pathological variations in sleep physiology may necessitate adaptation. Furthermore, steps must be taken to optimize stimulation parameters and targeting to maximize the therapeutic potential of these approaches. For patients with PD seeking to improve their sleep via these means, participation in clinical trials investigating TES sleep neuromodulation likely provide the safest and most informative path forward.

### 2.5 Auditory stimulation

Auditory stimulation, the use of time-synced auditory tones, has also been examined as a technique for the modulation of sleep physiology. Studies investigating the mechanisms of auditory or acoustic stimulation applied during sleep have demonstrated the potential to modulate both NREM [74] and REM sleep [75]. Like TES, the parameters of auditory stimulation are variable, though those used in sleep neuromodulation most commonly utilize phase-locked stimuli. This stimulus is understood to support and synchronize endogenous neural oscillations of sleep, thereby promoting sleep state maintenance. The enhancement of SWS via auditory stimulation has also been suggested to improve memory retention following nights of sleep with applied stimulus [75].

While studies of auditory stimulation in PD remain limited, ongoing research indicates that phase-synchronized auditory stimulation may be capable of increasing slow-wave activity [76]. This approach is particularly promising, as it can selectively target the intrinsic neural activity of SWS. However, its long-term effectiveness and generalizability remain uncertain, particularly in populations with altered neurophysiology, as is common PD. Future research should explore the sustained impact of auditory stimulation and evaluate its suitability in neurodegenerative conditions, where disrupted sleep architecture may impact responsivity to stimulation.

### 2.6 Light therapy

Light therapy is another non-invasive approach to sleep neuromodulation in PD. Light therapy refers to a wide range of treatments, though most relevant to PD sleep disruptions is the administration of light through a light box during wake. Most light boxes studied in PD emit white light (around 10,000 lm) [77], where a sunny day and an average indoor light provide 100,000 and 1,000 lm, respectively. It is hypothesized that daily administration of light therapy may help counteract circadian disturbances experienced by many patients with PD [78]. Patients are generally encouraged to administer this therapy on a daily basis in order to achieve and maintain therapeutic benefit. Previous studies have demonstrated the potential therapeutic impact of this approach, where patients with PD that underwent light therapy demonstrated improvements of roughly 30 % in daytime sleepiness, 20 % in subjective sleep quality, and reduction in overnight sleep disruptions [78], [79]. Blue light therapy is also currently under investigation in PD, as it may be more effective in influencing circadian function [77].

### 2.7 Nerve stimulation

Targeted nerve stimulation, including vagus nerve stimulation (VNS) and vestibular nerve stimulation (VeNS), have also shown to be capable of modulating sleep characteristics in clinical populations. VNS has been demonstrated to increase both TST as well as SWS duration in patients with epilepsy [80]. The effect of VNS on the autonomic nervous system is also relevant as it pertains to sleep quality, as applied stimulation increases parasympathetic tone, which may help lower physiological arousal [81] (Clancy et al., 2014). VeNS is relatively newer, but clinical trials have demonstrated a reduction in the severity of insomnia following application [82]. As research continues in this area, it will be beneficial to examine the potential therapeutic impact that these approaches may have on sleep in neurodegenerative disorders such as PD.

Hypoglossal nerve stimulation (HNS) is another method of targeted nerve stimulation that is growing in popularity for the treatment of obstructive sleep apnea in patients unable to tolerate continuous positive airway pressure (CPAP) [83]. HNS functions via direct stimulation of the hypoglossal nerve synchronized with inspiration to maintain airway patency during sleep. Treatment efficacy has been well established in the general public, though HNS has yet to be systematically studied in PD [84]. However, implication of the hypoglossal nerve in PD pathology suggests that HNS may be a promising option for the treatment of not just sleep disordered breathing but wider sleep disruption in PD.

### 2.8 Deep brain stimulation

Deep brain stimulation (DBS) is a well-established treatment for motor symptoms in advanced PD. Its effects on sleep are not yet fully understood, though research indicates that it can influence sleep on the patient level [85], [86], [87]. Improvements in sleep following DBS for PD may be largely derived from improved overnight motor symptoms. Furthermore, conventional DBS may actually disrupt sleep, particularly slow-wave sleep, as stimulation parameters remain unchanged overnight [34]. Therefore, attention has been directed towards methods such as adaptive DBS (aDBS), capable of modulating simulation in response to changes in cortical activity. Recent research has demonstrated the potential to detect and stage sleep using local field potentials collected from the DBS lead [88]. Closed loop algorithms trained on the cortical activity during deep N2 and N3 sleep have been capable of decreasing subcortical stimulation amplitude during SWS [86], which may allow for continued nocturnal DBS without concern for the disruption of sleep states.

Currently, there are no commercially available products for the specific treatment of sleep dysfunction in PD. Interventions such as DBS often result in the improvement of various sleep indices, but treatment is not indicated in less severe cases of PD. Non-invasive approaches have grown in both variety and understanding but have not yet demonstrated the efficacy required for results to be translated to clinical care. However, ongoing development of these approaches may hold broader implications for the treatment of PD sleep dysfunction by augmenting the current standard of care.

## 3 Conclusion

The progressive degradation of healthy sleep is central to the presentation and progression of Parkinson’s disease. A complex intertwining of neurodegeneration and sleep dysfunction contribute to disease progression and worsened quality of life, yet current treatment approaches do not detect or address the full spectrum of pathology and are not readily accessible to all patients. Emerging neurotechnological advancements are promising in this regard, offering novel avenues to build upon current pharmaceutical and behavioral treatment modalities. Leveraging these tools, in combination with comprehensive diagnostic monitoring of sleep, may pave the way for a therapeutic program capable of slowing disease progression in PD.

## CRediT authorship contribution statement

**Mason Dallegge:** Writing – review & editing, Writing – original draft, Visualization, Conceptualization. **Sanjana Murthy:** Writing – review & editing, Writing – original draft. **Don M. Tucker:** . **Emmanuel During:** Writing – review & editing. **Shannon O’Neill:** Writing – review & editing, Writing – original draft. **Rachel Fremont:** Writing – review & editing. **Joohi Jimenez-Shahed:** Writing – review & editing. **Allison C. Waters:** Writing – review & editing, Writing – original draft, Conceptualization.

## Declaration of competing interest

The authors declare the following financial interests/personal relationships which may be considered as potential competing interests: The following authors report no conflicts of interest: MD, AW, RF, SO, SJ, ED.

DT is an employee of the Brain Electrophysiology Laboratory Company and Neurosom, Inc., makers of the Wireless Interface Sensor Pod (WISP) sleep therapy system.

JJS has received consulting fees from Medtronic, BlueRock, Abbvie, Amneal, Teva and TreeFrog; has received research funds from ONO, Sage, and Annovis; serves on the DSMB for Biohaven and Emalex; and the scientific advisory board for Photopharmics.

Figures were generated with Biorender.com: Waters, A. (2025) https://BioRender.com/ophiw8d; https://BioRender.com/xk8kuzi.
